# Candidate tumor suppressor ZNF154 suppresses invasion and metastasis in NPC by inhibiting the EMT via Wnt/β-catenin signalling

**DOI:** 10.18632/oncotarget.20479

**Published:** 2017-08-24

**Authors:** Ying Hu, Min-Fang Qi, Qian-Lan Xu, Xiang-Yun Kong, Rui Cai, Qiu-Qiu Chen, Hua-Ying Tang, Wei Jiang

**Affiliations:** ^1^ Department of Radiation Oncology, Affiliated Hospital of Guilin Medical University, Guilin 541001, P.R. China; ^2^ Department of Pathology, The 181st Hospital of People's Liberation Army, Guilin 541001, P.R. China; ^3^ Department of Oncology, Xianning Central Hospital, The First Affiliated Hospital of Hubei University of Science and Technology, Xianning 437100, P.R. China

**Keywords:** nasopharyngeal carcinoma, zinc finger protein 154, candidate tumor suppressor, methylation

## Abstract

**Background:**

Nasopharyngeal carcinoma (NPC) is especially prevalent in southeast Asia and southern China, but its molecular mechanisms remain poorly characterized. DNA methylation is associated with initiation and progression of tumors, including NPC. Through a genome-wide DNA methylation screening approach, we discovered ZNF154, but its methylation status and roles in NPC have not been investigated.

**Methods:**

The methylation status of ZNF154 in NPC was detected with Methylation specific-PCR (MSP) and Quantitative Sequenom MassARRAY. The invasion and migration capacities were examined by wound healing and transwell invasion assays. The role of ZNF154 in NPC metastasis was clarified with experimental metastasis assay *in vivo*. Western blotting analysis was used to investigate protein changes followed by ZNF154 over-expression. Kaplan-Meier analysis was performed to determine the association between ZNF154 methylation and prognosis in NPC.

**Results:**

Compared to immortalized nasopharyngeal tissues and cells, ZNF154 expression was frequently downregulated in NPC tissues and cell lines due to promoter methylation. Demethylation treatment with 5-aza-2-deoxycytidine (5-Aza) restored ZNF154 expression in NPC cell lines. Ectopic overexpression of ZNF154 in NPC cells inhibited cell migration and invasion *in vitro* and lung nodule formation in an *in vivo* tumor metastasis assay. Mechanistic investigations suggested ZNF154 inhibits Wnt/β-catenin signalling pathway activation and prevents the EMT in NPC. Furthermore, Kaplan-Meier analysis showed hypermethylation of the ZNF154 promoter was associated with significantly poorer disease-free survival (P = 0.032) and distant metastasis-free survival (P = 0.040) among patients with locoregionally advanced NPC.

**Conclusions:**

Taken together, these findings define a novel role for ZNF154 as a tumor suppressor in NPC.

## INTRODUCTION

Nasopharyngeal carcinoma (NPC) is a malignant tumor of the nasopharyngeal epithelium that is most prevalent in Southeast Asia, particularly Southern China [1]. Developments in radiotherapy and combined chemo-radiotherapy have significantly improved 5-year overall survival for NPC [2]. However, NPC cells can easily metastasize to distant organs, and distant metastasis is the major pattern of treatment failure [3]. Therefore, it is crucial to better understand the molecular mechanisms driving tumor metastasis in order to develop more effectively treatments for NPC.

Zinc finger proteins (ZFPs) are the largest family of transcription factors in the human genome, and bind to gene promoters via zinc finger domains to activate or repress gene expression [4]. About one third of ZFPs contain a Kruppel-associated box (KRAB) domain [5]. KRAB-containing zinc finger proteins (KRAB-ZFP) are implicated in the regulation of multiple cellular processes including differentiation, proliferation, the cell cycle and apoptosis [6]. Moreover, aberrant inactivation of KRAB-ZFPs has been associated with abnormal gene expression and tumorigenesis [7]. Therefore, characterization of the functional significance of the KRAB-ZFPs family members in NPC may extend our current knowledge of the molecular mechanisms of nasopharyngeal carcinogenesis, and provide insight to identify novel potential targets for the diagnosis and treatment of NPC. Through a genome-wide DNA methylation screening approach in our previous study [8], a candidate KRAB-ZFP member, zinc finger protein 154 (ZNF154), was identified to play an important role in the occurrence and development of NPC.

In this study, we characterized ZNF154 expression, promoter methylation and the associations between these factors in NPC. Functional *in vitro* and *in vivo* assays were performed using ZNF154-overexpressing NPC cell lines to characterize the biological effects of ZNF154 in nasopharyngeal carcinogenesis. Furthermore, the mechanism by which ZNF154 inhibits tumor metastasis in NPC was identified.

## RESULTS

### ZNF154 is frequently downregulated by promoter methylation in NPC tissues and cell lines

**Figure 1:**
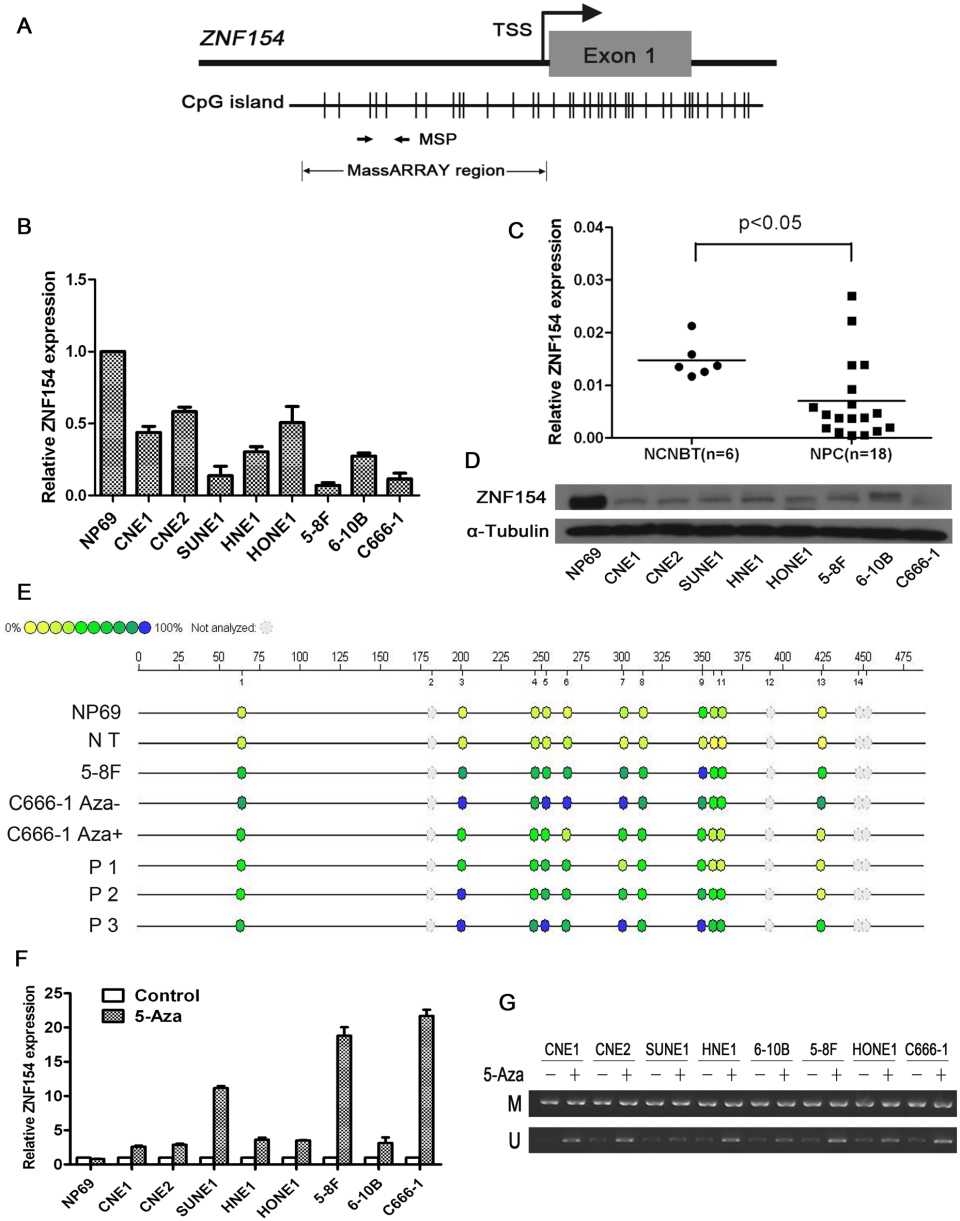
ZNF154 is downregulated by promoter methylation in nasopharyngeal carcinoma (NPC) **(A)** CpG islands in ZNF154. The transcription start site (TSS) is indicated by a curved arrow. The regions analysed by methylation specific PCR (MSP) and MassARRAY are shown. **(B)** Relative ZNF154 expression in normal nasopharyngeal NP69 cells and NPC cell lines. Glyceraldehyde 3-phosphate dehydrogenase (*GAPDH*) was used as the endogenous control. **(C)** Relative ZNF154 expression in NPC tissues and non-cancer nasopharyngitis biopsy tissues (NCNBT). **(D)** Western blotting analysis of ZNF154 protein expression in NPC cell lines and NP69 cells. **(E)** Quantitative MassARRAY methylation analysis of ZNF154 in NP69 cell lines, normal tissues (NT), NPC cell lines and NPC tissue specimens (P). **(F)** Demethylation treatment with 5-Aza restored the expression of ZNF154 in NPC cell lines. **(G)** MSP analysis of the methylation status of ZNF154 after demethylation treatment of NPC cell lines; M, methylated allele; U, unmethylated allele.

Quantitative RT-PCR revealed ZNF154 was expressed in the normal nasopharyngeal cell line NP69 and non-cancer nasopharyngitis biopsy tissues, whereas absent or reduced expression of ZNF154 was observed in the eight NPC cell lines and human NPC tissues (Figure 1B–1D). The methylation status of ZNF154 was assessed using the Sequenom MassARRAY platform. The ZNF154 promoter was frequently hypermethylated in NPC tissues and cell lines, with low levels of methylation observed in NP69 cells and the normal nasopharyngeal tissues (Figure 1E). To further clarify whether promoter methylation directly mediates downregulation of ZNF154 in NPC, the eight NPC cell lines and NP69 cells were treated with the demethylation agent 5-Aza. The expression of ZNF154 was restored in all NPC cell lines by demethylation treatment (Figure 1F and 1G), indicating that promoter methylation contributes to downregulation of ZNF154 in NPC.

### ZNF154 suppresses NPC cell migration and invasion *in vitro*

**Figure 2:**
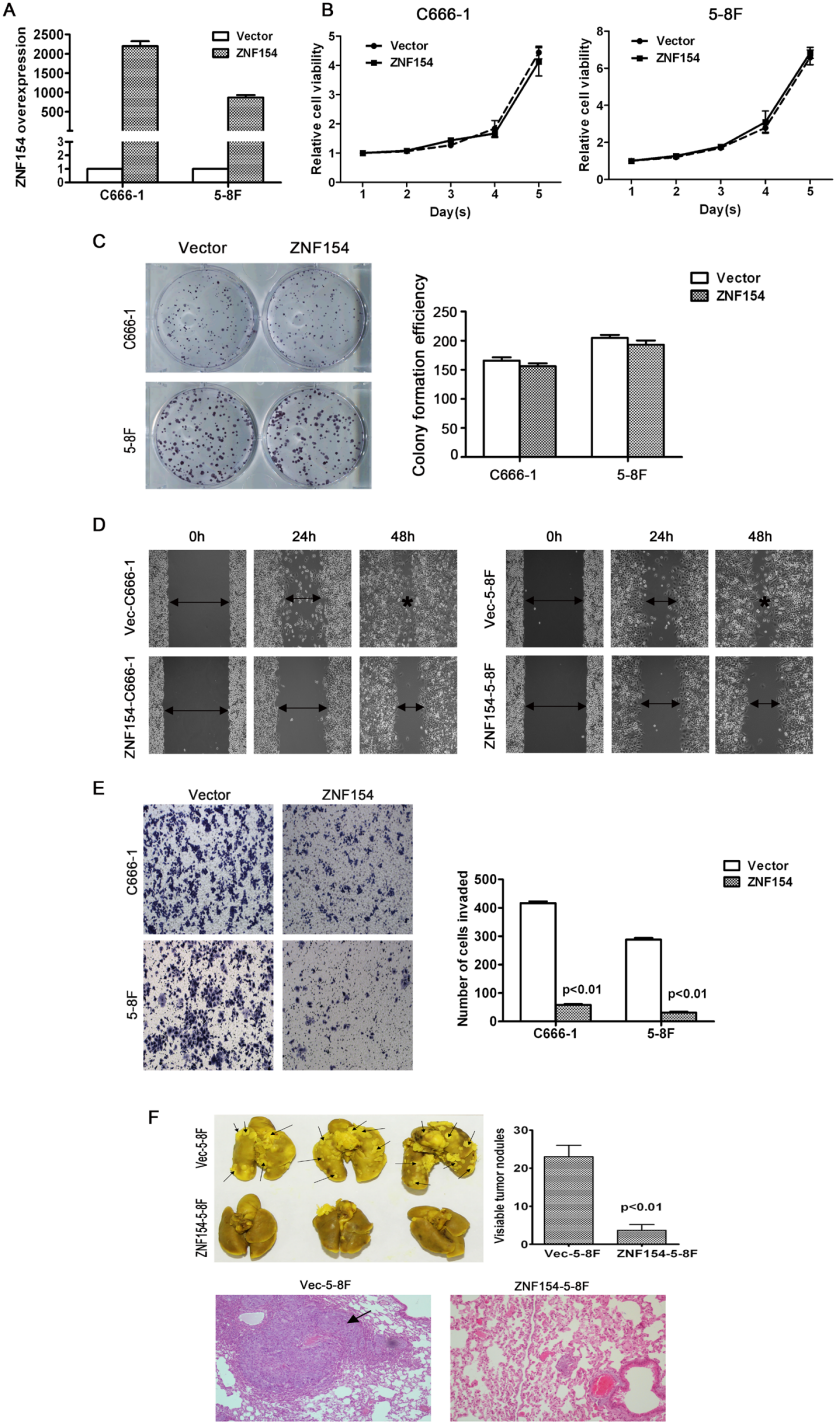
*In vitro* and *in vivo* analysis of the tumor suppressor function of ZNF154 in nasopharyngeal cancer (NPC) **(A)** Ectopic overexpression of *ZNF154* in C666-1 and 5-8F cells. **(B)** ZNF154 did not suppress the viability of C666-1 or 5-8F cells. **(C)** Overexpression of ZNF154 did not significantly affect NPC cell line growth or colony formation. **(D)** Overexpression of ZNF154 inhibited NPC cell migration in the wound healing assay. **(E)** Overexpression of ZNF154 inhibited NPC cell invasion in the Matrigel invasion assay. **(F)** Representatives of lungs excised from nude mice at 8 weeks after injection of Vec-5-8F or ZNF154-5-8F cells. Representative images of lung tumor nodules. Metastatic nodules are indicated by arrows. Representative images of H&E staining of the lung cancer tissues of mice injected with Vec-5-8F or ZNF154-5-8F cells.

In order to investigate whether ZNF154 is essential for the growth, migration and invasion of NPC cells, ZNF154 was cloned into an expression vector and transfected into two NPC cell lines, C666-1 and 5-8F (Figure 2A). Ectopic overexpression of ZNF154 did not inhibit cell viability or colony formation (*P* > 0.05, Figure 2B and 2C). The wound healing assay demonstrated overexpression of ZNF154 significantly inhibited cell migration compared to vector control cells (Figure 2D). The Transwell invasion assay showed overexpression of ZNF154 significantly reduced invasive ability compared to cells transfected with the empty vector (*P* < 0.05, Figure 2E). Taken together, these findings indicate restoring the expression of ZNF154 suppressed NPC migration and invasion *in vitro*.

### ZNF154 suppresses tumor metastasis *in vivo*

An *in vivo* experimental metastasis assay was established to clarify the role of ZNF154 in NPC metastasis; 5-8F cells, which have a high metastatic rate, were stably transfected to overexpress ZNF154 or vector control, and injected into nude mice via the tail vein. After 8 weeks, the mice were sacrificed and formation of metastatic nodules in the lung was assessed. Mice injected with cells overexpressing ZNF154 possessed significantly fewer lung nodules than mice injected with the vector control cells (*P* < 0.01, Figure 2F), and H&E staining verified these findings (Figure 2F), indicating that restoring the expression of ZNF154 inhibited tumor metastasis *in vivo*.

### ZNF154 inhibits metastasis in NPC by regulating Wnt/β-catenin signalling to suppress the epithelial mesenchymal transition

**Figure 3:**
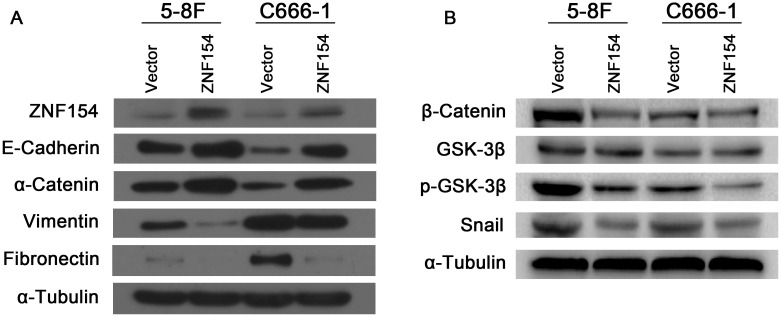
ZNF154 affects expression of epithelial-mesenchymal transition (EMT)-related proteins in nasopharyngeal cancer cell lines **(A)** Western blot analysis revealed overexpression of ZNF154 upregulated E-cadherin and α-catenin and downregulated vimentin and fibronectin. **(B)** Overexpression of ZNF154 downregulated β-catenin, p-GSK-3β and Snail in C666-1 and 5-8F cells; α-tubulin was used as a loading control.

The epithelial mesenchymal transition (EMT) is associated with tumor cell invasion and metastasis in most carcinomas. Thus, we investigated whether ZNF154 suppresses tumor metastasis via regulating the EMT. Western blotting showed overexpression of ZNF154 upregulated the epithelial markers E-cadherin and α-cadherin and downregulated the mesenchymal markers vimentin and fibronectin in C666-1 and 5-8F cells (Figure 3A) (see the intensity of bands in the Supplementary Table 1), suggesting expression of ZNF154 suppresses NPC cell metastasis by inhibiting an EMT phenotypic transition.

Wnt/β-catenin, TGF-β, Notch and Hedgehog signalling are major pathways implicated in the EMT [9]. Emerging data suggests the Wnt/β-catenin pathway plays a role in carcinogenesis in NPC [10, 11]. The Wnt/β-catenin signalling pathway was examined to gain insight into how ZNF154 regulates expression of EMT markers. Western blotting revealed overexpression of ZNF154 in 5-8F or C666-1 cells downregulated β-catenin, p-GSK-3β and Snail (Figure 3B) (see the intensity of bands in the Supplementary Table 2). Therefore, ZNF154 may inhibit the EMT by modulating inactivation of the Wnt/β-catenin signalling pathway.

### Promoter methylation of ZNF154 is associated with poor survival in locoregionally advanced NPC

**Figure 4:**
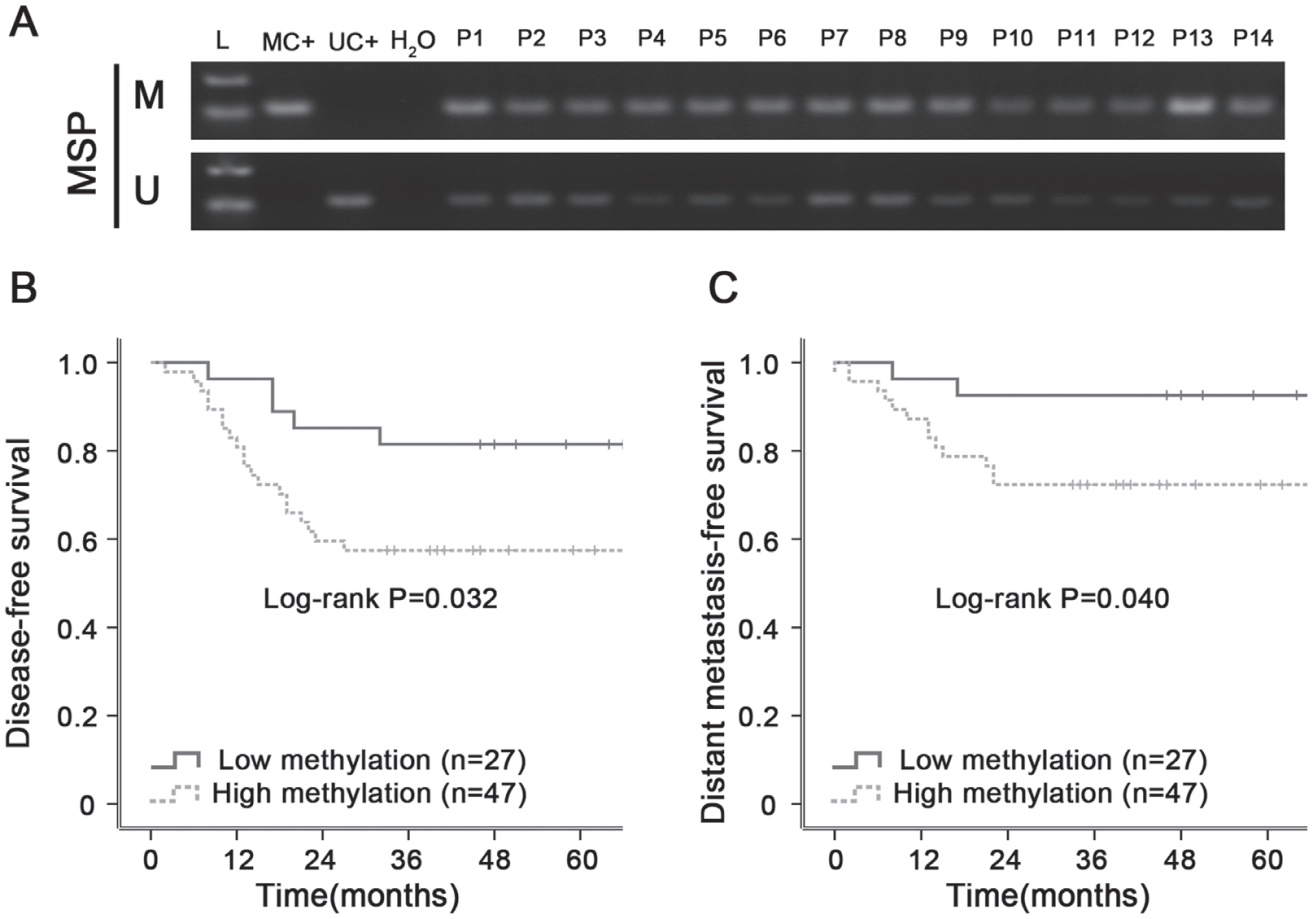
Hypermethylation of ZNF154 is associated with poorer survival outcomes in primary human nasopharyngeal carcinoma (NPC) **(A)** Methylation-specific PCR (MSP) analysis of ZNF154 promoter methylation in NPC tissue specimens. M, methylated; U, unmethylated; L, marker; MC+, methylated positive control, UC+, unmethylated positive control; water serve as negative control for MSP. **(B, C)** Kaplan-Meier survival curves showing patients with locoregionally advanced NPC and ZNF154 hypermethylation had poorer disease-free survival (B) and distant metastasis-free survival (C) than patients with ZNF154 hypomethylation.

Retrospective analysis of patient samples revealed the methylation status of the ZNF154 promoter was significantly associated with survival outcomes in NPC. In the study, we identified differential DNA methylation levels through quantified the intensities of bands for MSP. The intensity values of M band and U band were generated for each patient. Only patients with less than the median intensity values of M bands and greater than U bands median intensities were separated into hypomethylation group, other patients were separated into hypermethylation group. MSP revealed ZNF154 was frequently methylated in human NPC tissues (Figure 4A). Seventy-four patients with advanced stage NPC were followed up for ≥ 5 years; 15 cases developed metastases, 14 had recurrent disease and 17 died. Kaplan-Meier analysis demonstrated patients with ZNF154 hypermethylation had significantly poorer disease-free survival (DFS, 3-year DFS rates, 57% vs. 82%, *P* = 0.032) and distant metastasis-free survival (DMFS, 3-year DMFS rates, 72% vs. 92%, *P* = 0.040) than patients with ZNF154 hypomethylation (Figure 4B and 4C). Thus, ZNF154 methylation status may have prognostic value for survival outcomes in NPC, especially metastasis in patients with locoregionally advanced disease.

## DISCUSSION

This is the first investigation of the frequency and importance of a ZNF154 epigenetic alteration in NPC. We report the ZNF154 promoter is frequently methylated in NPC tissues, but not the adjacent normal nasopharyngeal tissues. Moreover, hypermethylation plays a pivotal role in transcriptional silencing of ZNF154 in NPC, while restoring the expression of ZNF154 inhibited NPC cell migration, invasion and metastasis and suppressed the EMT, indicating ZNF154 functions as a putative tumor suppressor in NPC.

DNA methylation is a major mechanism that leads to inactivation of tumor suppressor genes in many types of cancer [12], including NPC [13, 14]. Hypermethylation of ZNF154 has been reported in breast [15], lung [16], hepatocellular [17], ovarian [18], renal [19] and prostate cancer [20]. Similarly, this study indicates the ZNF154 promoter is frequently methylated in human primary NPC cells and tissues. Furthermore, methylation mediates transcriptional silencing of ZNF154 as demethylation treatment restored its expression.

The tumor-suppressive effect of ZNF154 was assessed both *in vitro* and *in vivo*. Ectopic expression of ZNF154 effectively suppressed NPC cell migration and invasion *in vitro* and reduced metastatic tumor nodule formation in the lungs *in vivo*. These findings suggest ZNF154 is a novel tumor suppressor that inhibits migration, invasion and metastasis in NPC. Further analysis revealed the tumor-suppressive function of ZNF154 was closely associated with the EMT. The EMT is characterized by phenotypic changes, such upregulation of mesenchymal markers and downregulation of epithelial markers [21], and is closely related to tumor cell migration, invasiveness and metastasis [22–24]. Overexpression of ZNF154 upregulated the epithelial markers E-cadherin and α-catenin and downregulated the mesenchymal markers vimentin and fibronectin in NPC cell lines. E-cadherin and α-catenin mediate cell-cell adhesion and maintain cell-to-cell contact, which prevents tumor cell invasion and metastasis [23]. Thus, these observations indicate loss of ZNF154 expression due to promoter hypermethylation promotes cell invasion and metastasis by inducing EMT-like changes in NPC.

Mechanistically, western blotting analysis revealed the Wnt/β-catenin signalling pathway may be involved in the ability of ZNF154 to inhibit the EMT. Aberrant activation of the Wnt/β-catenin signalling pathway is closely associated with cancer initiation, progression and metastasis [25, 26]. Moreover, the Wnt/β-catenin signalling pathway may influence the expression of E-cadherin [27]. Overexpression of ZNF154 in 5-8F and C666-1 cells downregulated β-catenin, p-GSK-3β and Snail, leading to upregulation of E-cadherin. Hence, we conclude that ZNF154 functions as a tumor suppressor to prevent the EMT by inhibiting Wnt/β-catenin signalling pathway activation.

DNA methylation is recognized as a useful marker for early diagnosis and predicting prognosis in multiple types of cancer [13, 28]. This study indicates ZNF154, which appears to function as a tumor suppressor to inhibit Wnt/β-catenin signalling and prevent tumor cell invasion and metastasis, is frequently downregulated due to promoter hypermethylation in NPC. Therefore, ZNF154 promoter methylation status may have potential as prognostic biomarker of tumor progression or metastasis in NPC. We assessed the methylation status of the ZNF154 promoter in human NPC tissues by MSP analysis. ZNF154 hypermethylation was associated with significantly poorer DFS and DMFS in patients with locoregionally advanced NPC. This finding suggests reversing DNA methylation of the ZNF154 promoter could represent a potential target for anticancer therapy.

Taken together, this study demonstrates that ZNF154 is commonly downregulated or silenced by promoter methylation in NPC. ZNF154 can suppress NPC cell invasion and metastasis through inhibiting Wnt/β-catenin signalling pathway activation and suppressing the EMT. Moreover, hypermethylation of the ZNF154 promoter is associated with significantly poorer survival in patients with locally advanced NPC, who are at high risk of metastasis. Therefore, methylation of the ZNF154 promoter could represent a promising candidate biomarker for predicting prognosis and provide a novel therapeutic target for NPC.

## MATERIALS AND METHODS

### Cell culture

Human nasopharyngeal cancer cell lines (CNE1, CNE2, HNE1, SUNE1, HONE1, C666-1, 5-8F, 6-10B) and the immortalized human nasopharyngeal epithelial cell line NP69 were obtained from Sun Yat-sen University Cancer Centre. All cancer cell lines were cultured in RPMI-1640 (Gibco, Carlsbad, CA, USA) supplemented with 10% foetal bovine serum (FBS) at 37°C in a 5% CO2 atmosphere. NP69 cells were incubated in Keratinocyte/serum-free medium supplemented with bovine pituitary extract (Invitrogen, Carlsbad, CA, USA). 293FT cells were cultured in DMEM (Invitrogen) supplemented with 10% FBS.

### Patient samples

Seventy-four freshly frozen NPC tissues and six non-cancer nasopharyngitis biopsy tissues (NCNBT) were obtained from the primary site before treatment from patients treated at the Affiliated Hospital of Guilin Medical University and 181st Hospital of People's Liberation Army. All 74 patients were confirmed to have primary undifferentiated non-keratinizing NPC. Median age was 46 (range, 30-73) years; 53 patients were male and 21 were female; 52 cases had stage III, 17 had stage IVa and 5 had stage IVb NPC. Written informed consent was obtained from all participants. Tissue collection and analyses were approved by the Institutional Review Board of both centres.

### Total RNA extraction, reverse transcription and quantitative reverse transcription-PCR

Total RNA was extracted from frozen NPC tissues and cell lines using Tri-Reagent (Invitrogen) and 2 μg was reverse transcribed. Quantitative PCR was performed on a Bio-Rad CFX96 sequence detection system (Bio-Rad Laboratories Inc., Hercules, CA, USA) using Platinum SYBR Green qPCR SuperMix-UDG reagent (Life Technologies, Carlsbad, USA) at 95°C for 10 min, followed by 45 cycles of 95°C for 30 s, annealing at 60°C for 60 s and elongation at 72°C for 7 min. Human glyceraldehyde-3-phosphate dehydrogenase (*GAPDH)* was used as the endogenous control gene [29]. Relative expression was determined via the 2^-ΔΔCT^ method. The primer sequences are listed in Table 1.

**Table 1:** PCR primer sequences

Primer	Sequence (5′-3′)
Quantitative reverse-transcription PCR	
Forward primer	CAACTCAGGGCACTGTGACC
Reverse primer	GGCTCTGCCCACATTGCTTC
Methylation-specific PCR	
Methylated forward primer	TAAGTTATTTTTTATCGTTTAGCGT
Methylated reverse primer	TAATATAATTTTCATAAATCCCGAA
Unmethylated forward primer	GGATAAGTTATTTTTTATTGTTTAGTGT
Unmethylated reverse primer	TAATATAATTTTCATAAATCCCAAA
Sequenom MassARRAY PCR	
Forward primer	TTGAATGGTTTTATGGAATGATGTA
Reverse primer	CACACCTCAAAAAAACTAAAATAACC

### DNA bisulphite modification

Genomic DNA was extracted from the freshly-frozen tissue samples using All Prep DNA/RNA Mini Kit (Qiagen, Hilden, Germany) according to the manufacturer’s recommendations. DNA quality and quantity were assessed using an Agilent 2100 Bioanalyzer (Agilent Technologies, Palo Alto, USA) and NanoDrop ND 1000 spectrophotometer (Thermo Scientific, Hudson, NH, USA). DNA was then bisulphite-converted using EZ DNA methylation kit (Zymo Research, Orange, CA, USA) according to the manufacturer’s recommendations.

### Treatment with 5-Aza-2′-deoxycytidine

To assess whether demethylation restored ZNF154 expression, NPC cells were treated with 10 μmol/L 5-aza-2′-deoxycytidine (5-Aza; Sigma, Santa clara, CA, USA) for 3 days, then total DNA and RNA were extracted; the 5-aza-2′-deoxycytidine containing medium was replaced every 24 h.

### Methylation specific-PCR

Total DNA was extracted from clinical tissue samples and NPC cell lines using the phenol-chloroform method. Genomic DNA was subjected to bisulphite treatment and analysed by methylation specific-PCR at 95°C for 5 min, followed by 35 cycles of 95°C for 30 s, 60°C for 60 s and 72°C for 30 s, and final extension at 72°C for 5 min. The sequences of the methylated and unmethylated DNA primers are listed in Table 1. Each PCR reaction contained 300 ng sodium bisulphite-modified DNA, 10× MSP PCR buffer, 2.5 mM deoxyribonucleotide triphosphates (dNTPs) and 1 U Polymerase (Roche, Penzberg, Germany). The PCR products were analysed on 2% agarose gels.

### Quantitative sequenom MassARRAY methylation analysis

The Sequenom MassARRAY platform (Oebiotech, Shanghai, China) was utilized to quantitatively analyse the methylation status of the ZNF154 gene promoter. PCR primers (Table 1) were designed using Methprimer (http://www:epidesigner.com). The PCR mixtures were pre-heated for 4 min at 94°C, followed by 45 cycles of 94°C for 20 s, 56°C for 30 s and 72°C for 60 s, then final extension at 72°C for 3 min. PCR products were incubated with Shrimp Alkaline Phosphatase following the manufacturer’s protocol. After *in vitro* transcription and RNaseA digestion, small RNA fragments with CpG sites were acquired for the reverse reaction. The methylation ratios of the products were calculated using Epityper software Version 1.0 (Sequenom, San Diego, CA, USA).

### Construction of ZNF154 expression vector and stably transfected cell lines

The full-length open reading frame (ORF) of human ZNF154 cDNA was amplified by PCR and cloned into the lentiviral vector pSin-EF2-puromycin (Addgene, Cambridge, MA, USA). The primers of ZNF154 were Bgl II- sense: 5' GAAGATCTGCCACCATGGCAGCGGCCACTCTGAGG 3' and Spe I-sense: 5' GGACTAGTTTATCGACTATGAATTCTCTGATG 3'. The vector control pSin-EF2 or pSin-EF2-ZNF154 plasmid was co-transfected with PMD2.G and PSPAX2 plasmid into 293FT cells using the Calcium Phosphate method. The supernatants were collected 24h after transfection and used to infect 5-8F and C666-1 cells. Stably transfected cells were selected with puromycin and validated by quantitative RT-PCR analysis.

### Cell proliferation assay

For the 3-(4,5-dimethy(thiazol-2-yl)-2,5-diphenyltetrazolium bromide (MTT) assay, 1 × 10^3^ vector control or ZNF154-overexpressing cells/well were seeded into 96-well plates, incubated for 1, 2, 3, 4 or 5 days, washed with PBS, 20 μL of 5 mg/mL MTT solution (Sigma) was added to each well, incubated for 4 h at 37°C, then 150 μL dimethyl sulfoxide (DMSO) added to dissolve formazan crystals. After shaking for 10 min at room temperature, absorbance values were measured at 490 nm.

### Colony formation and would healing assays

For the colony formation assay, 5-8F and c666-1 stable cell lines were plated of 400 cells per well in 6-well plates, incubated for 7 or 12 days and colonies containing > 50 cells were counted.

For the wound healing assay, vector control or ZNF154-overexpressing cells were seeded into 6-well plates, cultured until confluent (> 95%), starved in serum-free medium for 24 h, then the monolayers were scratched using a sterile tip to form an artificial would, and closure of the scratch was observed at 0, 24 and 48 h by light microscopy (Olympus, Tokyo, Japan).

### Transwell invasion assay

Invasive ability was assessed using Matrigel invasion chambers (Corning, Corning, NY, USA). Briefly, vector control or ZNF154-overexpressing cells (1 × 10^5^) were seeded onto the upper surface of polycarbonate membranes (8-μm pore size) coated with Matrigel (BD Biosciences) in serum-free-medium. RPMI-1640 supplemented with 10% FBS was added to the lower chamber, the plates were incubated for 24 h, then cells that had invaded to the lower membrane were fixed in methanol, stained with 0.1% crystal violet and counted under an inverted light microscope.

### Western blot analysis

Western blot analysis was performed according to standard procedures [30] using a primary antibody and secondary antibody (Cell Signaling Technology, Boston, MA); a α-tubulin antibody (Cell Signaling Technology) was used as a loading control. Signals were detected using the enhanced chemiluminescence substrate kit (Abcam, Cambridge, UK).

### *In vivo* metastasis assay

Female nude mice (4-weeks old) were purchased from Guangdong Experimental Animal Center. All protocols were approved by the Institutional Animal Care and Use Committee. Vector control or ZNF154-overexpressing 5-8F cells (1 × 10^6^) in 200 μL PBS were injected into nude mice via the tail vein. After 8 weeks, the mice were euthanized and their lungs were fixed, embedded in paraffin, and 5 μm-thick sections were subjected to H&E staining and examined by light microscopy.

### Statistical analysis

Statistical analysis was conducted using SPSS 18.0 (SPSS Inc., Chicago, IL, USA). All data are expressed as mean ± SD. Differences between two independent groups were examined using the Student’s *t*-test. Survival curves were generated using the Kaplan-Meier analysis and compared using the log-rank test. *P* < 0.05 was considered significant.

## SUPPLEMENTARY MATERIALS TABLES


