# Supplementary material for: Candidate tumor suppressor ZNF154 suppresses invasion and metastasis in NPC by inhibiting the EMT via Wnt/β-catenin signalling
Source: Oncotarget. 2017 Aug 24;8(49):85749–58. doi: 10.18632/oncotarget.20479 (PMC5689643; doi:10.18632/oncotarget.20479)
Supplement: Supplementary file 1 [file oncotarget-08-85749-s001.pdf]

Candidate tumor suppressor ZNF154 suppresses invasion and metastasis in NPC by inhibiting the EMT via Wnt/β-catenin signalling

SUPPLEMENTARY MATERIALS

Supplementary Table 1: Comparisons of the intensity of bands on the western blot in Figure 3A

	5-8F		C666-1	
	Vector	ZNF154	Vector	ZNF154
ZNF154	1	3.9292	1	3.7145
E-Cadherin	1	1.3840	1	2.6657
α-Catenin	1	1.4324	1	2.0908
Vimentin	1	0.2403	1	0.9570
Fibronectin	1	0.0689	1	0.0632

Supplementary Table 2: Comparisons of the intensity of bands on the western blot in Figure 3B

	5-8F		C666-1	
	**Vector**	**ZNF154**	**Vector**	**ZNF154**
β-Catenin	1	0.5311	1	0.7714
GSK-3β	1	1.0611	1	1.1050
p-GSK-3β	1	0.6696	1	0.4902
Snail	1	0.6899	1	0.5587
